# Absence of Rhythm Benefit on Speech in Noise Recognition in Children Diagnosed With Auditory Processing Disorder

**DOI:** 10.3389/fnins.2020.00418

**Published:** 2020-05-05

**Authors:** Christos Sidiras, Vasiliki Vivian Iliadou, Ioannis Nimatoudis, Doris-Eva Bamiou

**Affiliations:** ^1^Clinical Psychoacoustics Lab, 3rd Department of Psychiatry, Neuroscience Sector, Medical School, Aristotle University of Thessaloniki, Thessaloniki, Greece; ^2^Faculty of Brain Sciences, UCL Ear Institute, University College London, London, United Kingdom; ^3^Hearing & Deafness Biomedical Research Centre, National Institute for Health Research, London, United Kingdom

**Keywords:** auditory processing disorder, hearing, neural entrainment, dynamic attending theory, rhythm, cognition

## Abstract

Auditory processing disorder (APD) is a specific deficit in the processing of auditory information along the central auditory nervous system. It is characterized mainly by deficits in speech in noise recognition. APD children may also present with deficits in processing of auditory rhythm. Rhythmic neural entrainment is commonly present in perception of both speech and music, while auditory rhythmic priming of speech in noise has been known to enhance recognition in typical children. Here, we test the hypothesis that the effect of rhythmic priming is compromised in APD children, and further assessed for correlations with verbal and non-verbal auditory processing and cognition. Forty APD children and 33 neurotypical ones were assessed through (a) WRRC, a test measuring the effects of rhythmic priming on speech in noise recognition, (b) a battery of auditory processing tests, commonly used in APD diagnosis, and (c) two cognitive tests, assessing working memory and auditory attention respectively. Findings revealed that (a) the effect of rhythmic priming on speech in noise recognition is absent in APD children, (b) it is linked to non-verbal auditory processing, and (c) it is only weakly dependent on cognition. We discuss these findings in light of Dynamic Attention Theory, neural entrainment and neural oscillations and suggest that these functions may be compromised in APD children. Further research is needed (a) to explore the nature of the mechanics of rhythmic priming on speech in noise perception and why the effect is absent in APD children, (b) which other mechanisms related to both rhythm and language are also affected in this population, and (c) whether music/rhythm training can restore deficits in rhythm effects.

## Introduction

Auditory processing disorder (APD) is defined as a specific deficit in the processing of auditory information along the central auditory nervous system, including bottom–up and top–down neural connectivity (Iliadou et al., 2017) and is currently classified in the international statistical classification of diseases and related health problems, 10th edition (ICD-10) as H93.25. APD is linked to functional abnormalities and lesions beyond the cochlea (Musiek et al., 2005a; Gilley et al., 2016; Iliadou and Eleftheriadis, 2017). Children with APD present a wide range of auditory symptoms, including self-reported poor musical ability and/or appreciation of music, and, most commonly, impaired speech recognition in noise (American Speech-Language-Hearing Association [ASHA], 2005; American Academy of Audiology [AAA], 2010; British Society of Audiology, 2018). Recent studies have verified impairments in the perception and production of musical rhythm (Olakunbi et al., 2010; Scheffner et al., 2017; Sidiras et al., 2019).

Rhythm is commonly present in both speech and music (Lerdahl and Jackendoff, 1983; Arvaniti, 2009, 2012; Loukina et al., 2011). The syllabic utterance rate largely drives speech rhythm. This rate is quasi-isochronous and quite the same across languages ranging between 3 and 5 Hz (200–333 ms; Baltazani, 2007; Tilsen and Johnson, 2008; Wong et al., 2011; Tilsen and Arvaniti, 2013). The central auditory nervous system (CANS) has the ability to detect periodicities in any kind of auditory stimuli, syllabic rate included, a process called neural entrainment (Overath et al., 2015; Zoefel et al., 2018). The auditory stimuli evoke oscillatory responses in the CANS that are synchronized in terms of phase and frequency to the stimuli’s envelope, i.e., roughly speaking, to the stimuli’s fluctuations of intensity over time (for a descriptive model see Giraud and Poeppel, 2012). A legit hypothesis is that for the case of syllabic rate, this process is further facilitated by the specific frequency tuning of the auditory cortex, circa 4 Hz, i.e., its sensitivity peaks (Edwards and Chang, 2013; Picton, 2013; Overath et al., 2015). Neural entrainment does not abruptly stop when the rhythmic stimuli cease, but fades away progressively in terms of its amplitude, phase and rate (Henry and Herrmann, 2014), and modulates auditory perception as long as it is present (Giraud and Poeppel, 2012; Peelle and Davis, 2012; Andreou et al., 2015; Kosem et al., 2018).

According to the Dynamic Attending Theory (DAT; Jones and Boltz, 1989; Large and Jones, 1999; Bolger et al., 2013; Henry and Herrmann, 2014; Meltzer et al., 2015), neural evoked potentials described as oscillations result in oscillations in attention, in a way that stimuli temporally aligned with high neural excitability (i.e., when neural oscillations peak) are better processed compared to stimuli that are aligned with low neural excitability (i.e., when neural oscillation is at its lowest point), or when neural entrainment is not present at all. Note that the term ‘attending’ in DAT does not refer to the typical notion of attention, but rather to an enhancement in processing power (see Large and Jones, 1999, p. 33; Henry and Herrmann, 2014). DAT predictions have been supported by studies of auditory processing (Jones et al., 2002; Bolger et al., 2013, 2014; Sidiras et al., 2017) and visual processing (Escoffier and Tillmann, 2008; Escoffier et al., 2010; Miller et al., 2013).

Apart from the effects in the context of DAT, within which modulation in the perception of an event depends on its exact timing (i.e., moment of occurrence), research has revealed that speech syntax processing is enhanced by auditory rhythmic priming, independently of its exact timing (Przybylski et al., 2013; Kotz and Gunter, 2015). Przybylski et al. (2013) showed that children are better in grammaticality judgments for sentences when primed by a regular, rhythmic sequence, compared to irregular rhythmic sequence. Regular here means a sequence that creates a strong feel of ‘rhythmicity’ (i.e., it does make you move along with it), while in irregular sequences this feeling is less intense or even absent. Kotz and Gunter (2015) also present evidence for improved syntactic processing when speech is primed by rhythmic auditory stimuli.

Considering speech and musical rhythm, the former can be either synchronized or un-synchronized to the latter in a song. In the first case, speech is perceived as having a musical rhythm quality. A special case is rap music, in which the performer aligns the uttered syllables with high precision with the background music, creating an exaggerated effect of ‘rhythmic speech.’ The opposite happens when syllables are not aligned with underlying music. This is often apparent in karaoke sessions, where the performer is ‘out of rhythm.’ The part of the musical rhythm with which speech synchronization occurs, i.e., meter, is composed of events placed in specific moments in time (for a detail description of meter see Lerdahl and Jackendoff, 1983). As syllables last for a couple of 100s of milliseconds, their synchronization cannot be based on their whole duration, but rather performers choose a special moment. This special moment is the Perceptual Center (P-Center), and it is the moment the syllable is perceived to happen, where most of the ‘energy’ of the syllable is present (Morton et al., 1976; Villing, 2010; Sidiras et al., 2017). An easy way to get the intuition behind this notion is to utter ‘pa-pa’ and clap twice, once for each syllable. The points in time when claps occur, correspond to the P-Centers of the syllable. Thus, for the example of rap music, the synchronization of speech with musical rhythm occurs when the syllables’ P-centers are aligned with this rhythm’s metrical events. In unsynchronized speech the alignment is absent.

This work focuses on assessing differences in processing power, as indexed by speech in noise recognition, (a) when stimuli are aligned to rhythm vs. when no rhythm is present (referred to as ‘synchronized rhythm effect’), and (b) when stimuli are not aligned with rhythm vs. when no rhythm is present (referred to as ‘unsynchronized rhythm effect’). Particularly, the core interest of this study is how these effects differ in APD children compared to typically developing ones, and whether they correlate with auditory processing as measured in everyday clinic practice and cognition. In particular, we tested auditory working memory and auditory attention, as these processes have been linked to auditory processing (Weihing et al., 2014; Iliadou et al., 2018; Stavrinos et al., 2018). As the purpose of this study is not to investigate the relation of cognition in general and APD, we limited assessment of cognition to these specific measures.

The test used for measuring synchronized and unsynchronized rhythm effect was the Word Recognition-Rhythm Component test (WRRC; Sidiras et al., 2017). WRRC is a speech in noise recognition test in which each trial consists of a word primed by a short rhythmic or non-rhythmic beat sequence, and was created as part of basic APD research for measuring rhythm effects on speech in noise recognition for APD children. The beat sequence is a probe functioning as a neural entrainment inducer, and the word is the item whose perception (i.e., recognition) is supposed to be modulated by entrainment. In order to measure these effects, 3 measurements of speech recognition are made, i.e., one when synchronization occurs, one when synchronization does not occur, and one when rhythm is not present at all. In a previous study (Sidiras et al., 2017) a version of this test was implemented in typically developing children, and results revealed a positive synchronized rhythm effect (i.e., the word was better recognized when synchronized rhythm was present compared to no rhythm) and a partial unsynchronized rhythm effect (i.e., the first syllable was better recognized when un-synchronized rhythm was present compared to no rhythm). Note that the latter result is neither a prediction, nor disproves DAT, but rather expands on previous findings on enhanced syntax processing when preceded by auditory rhythm (Przybylski et al., 2013; Kotz and Gunter, 2015).

Auditory processing disorder children present deficits in rhythm perception (Isochrony Task; Sidiras et al., 2019). Thus, given the rhythm component in WRRC, we expect synchronized and unsynchronized rhythm effects to be less prominent or even absent in APD children. We also expect that rhythm effects on speech in noise recognition will correlate with processing of other kinds of auditory stimuli as well. Finally, we expect little or no correlation between rhythm effects and cognition for APD children, since there is evidence in this clinical group that rhythm perception and cognition do not correlate (Sidiras et al., 2019).

Specifically, the primary scientific hypothesis in this study is that a synchronized and a smaller unsynchronized rhythm effect will be present in typically developing children, but this effect will be smaller or absent in children with APD. A secondary hypothesis is that a. synchronized and unsynchronized rhythm effects will be correlated to results of auditory processing clinical tests and b. correlation between rhythm effect and cognition will be small or absent in APD children.

## Materials and Methods

### Participants

Forty children diagnosed with APD (mean age = 8.6, *SD* = 1.62, minimum = 6, maximum = 12) and 33 control children (mean age = 9.3, *SD* = 1.7, minimum = 6, maximum = 12) participated in this study. Children with APD were referred for listening and academic problems by speech pathologists and/or educators, and were diagnosed with APD in the Psychoacoustic Clinic in the AHEPA hospital of Thessaloniki. Diagnosis was made via a standardized clinic psychoacoustic test battery and was based on their performance deficit of 2 SDs from the mean of age-matched children on at least two tests on at least one ear, one of which was not verbal (Musiek et al., 2005a; American Academy of Audiology [AAA], 2010; Iliadou et al., 2017; British Society of Audiology, 2018). The inclusion criteria for control children were: (a) age appropriate writing skills (according to the teachers’ reports), (b) normal hearing thresholds, (c) Greek as first language, and (d) no known or suspected developmental disorder (according to both teachers and caregivers). We consider these criteria to satisfy the minimum requirements for a group to be characterized as typically developing.

All participants presented normal hearing sensitivity bilaterally as revealed by pure-tone audiometry (PTA) thresholds of 15 dB HL or better at all octave frequencies between 250 and 8000 Hz. ANOVA showed no statistically significant difference in age between groups (*F* = 3.6, *p* > 0.05). Parents and caregivers of both groups gave their written consent for participation in the study, in accordance with the World Medical Association’s Declaration of Helsinki.

### Testing

Auditory processing testing included the WRRC test (Sidiras et al., 2017; detailed description in the next section), a speech in noise test (SinB; Iliadou et al., 2009; Sidiras et al., 2016), two temporal resolution tests (Gaps-In-Noise, GIN; Musiek et al., 2005b; Shinn et al., 2009; and Random Gap Detection Test, RGDT; Keith, 2000), one dichotic listening test (Dichotic Digits, DD; Tzavaras et al., 1981), and two pattern sequencing processing tests (Pitch Pattern Sequence. PPS; Duration Pattern Sequence, DPS; Musiek, 1994). We also conducted two cognitive tests, i.e., a Working Memory test (Digit Span; Wechsler et al., 2003; Iliadou et al., 2018) and a sustained auditory attention test (SAA; Simos et al., 2007). All tests except for WRRC, and SAA, were delivered in a sound-treated booth via headphones (TDH-50P) at 60 dB HL through a CD player routed via a GSI 61 audiometer. WRRC was delivered through a laptop and headphones (Sennheiser, HD PRO 380 pro) in a sound-treated booth and SAA was delivered through loudspeakers, both at 60 dB HL, in a sound-treated booth. 60 dB HL was chosen as a comfortable hearing level at which most speech stimuli occur in everyday life.

Due to time constraints about half randomly selected APD children completed the SAA, GIN, DD, PPS, and DPS tests (see Table 1). For monaural tests (SinB, GIN, DD, PPS, and DPS) which yield two outputs each, one for each ear, the mean score was used for analysis. For typically developing children, the two temporal resolution tasks, the dichotic and the two pattern sequencing processing tests were not implemented.

**TABLE 1:** N and percentage of children that completed each test for APD and control group.

	**APD group (*n* = 40)**		**Control group (*n* = 33)**	
	***N***	**Percentage**	***N***	**Percentage**
WRRC	40	100	33	100
SinB	40	100	33	100
Digit Span	25	62.25	24	72.7
SAA	23	57.5	30	90.1
GIN	23	57.5	−	−
RGDT	31	77.5	−	−
DD	23	57.5	−	−
PPS	16	40	−	−
DPS	14	35	−	−

*WRRC, Word Recognition-Rhythm Component; SinB, Speech In Babble; SAA, Sustained Auditory Attention; GIN, Gaps-In-Noise; RGDT, Random Gap Detection Test; DD, Dichotic Digit; PPS, Pitch Pattern Sequence; DPS, Duration Pattern Sequence.*

#### Word-Recognition Rhythm Component (WRRC) Test

In each trial of WRRC a stimulus composed of a preceding beat sequence and the target bisyllabic word in noise, which is to be recognized, is binaurally presented. The WRRC test is specifically designed in such a way that SNR always corresponds approximately to the same point of the psychometric function across listeners. This is achieved by adjusting the Signal-to-Noise-Ratio (SRN) according to SinB SNR50% score (better ear), decreased (i.e., making recognition harder) by 4 dB. Decrement was inserted to counterbalance for binaural summation (Litovsky et al., 2006; Buss et al., 2008). The value of 4 dB was chosen after a pilot trial and error procedure.

**FIGURE 1:**
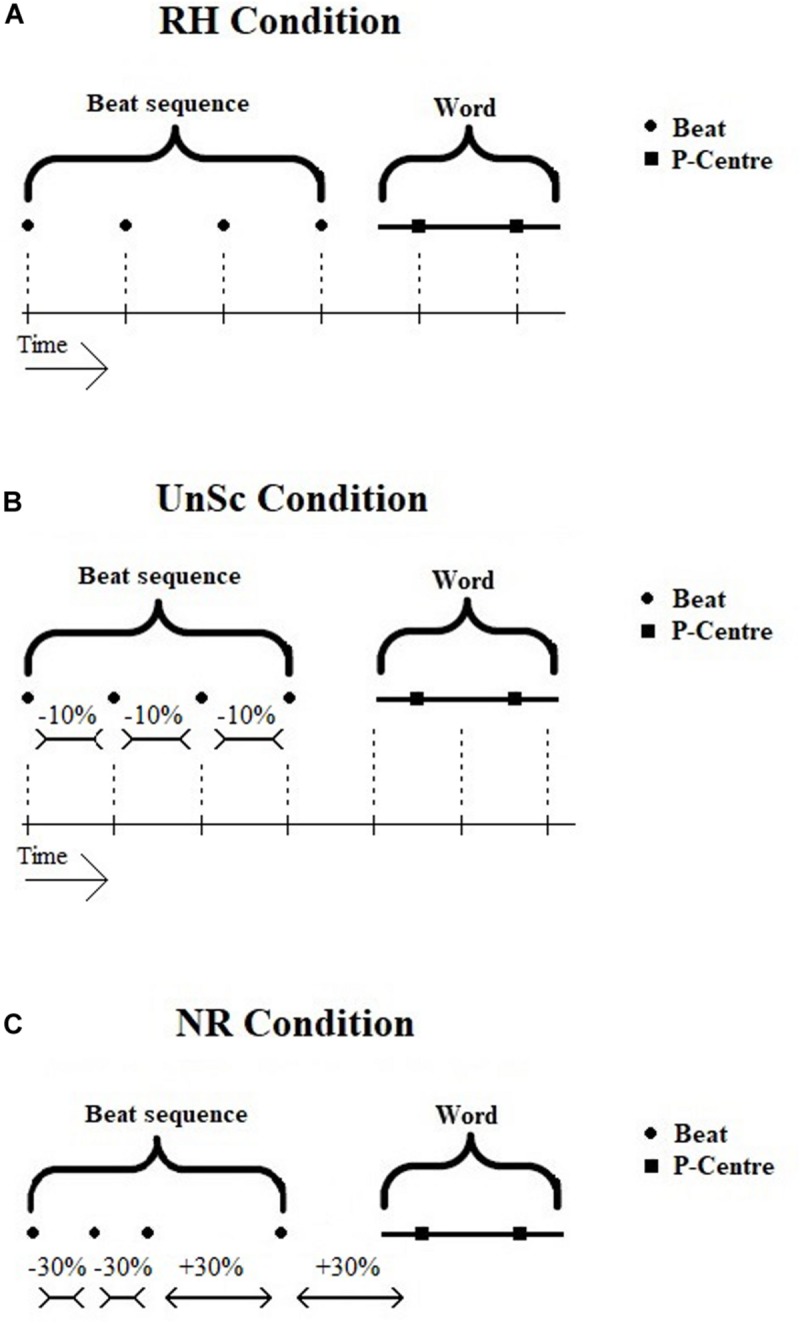
**(A–C)** Visual display of WRRC condition stimuli, i.e., beat sequence and word. Noise is not displayed in the figure. **(A)** RH Condition. Beat sequence and word is synchronized, all intervals are equal. **(B)** UnSc Condition. Word is not synchronized with beat sequence by shortening IPIs by 10%. Not all interval are equal. **(C)** NR Condition. Beat sequence is not isochronous by shortening and lengthening IPIs by 30%, hence no rhythm is present. RH, rhythm; UnSc, unsynchronized; NR, non-rhythm.

The preceding beat sequence functions as a probe and is either rhythmic (i.e., isochronous) or non-rhythmic (i.e., non-isochronous). In the former case, the beat sequence is supposed to cause neural entrainment and the following word’s P-Centers are either synchronized or unsynchronized to it (see Figure 1). These two conditions will be referred to from now on as ‘Rhythm Condition’ (RH) and ‘UnSynchronized Condition’ (UnSc) respectively. The condition in which the word is preceded by a non-rhythmic beat sequence does not invoke neural entrainment and will be referred to as ‘Non-Rhythm Condition’ (NR). The conditions were presented in three blocks respectively, in a random order. WRRC test was developed and executed on Matlab version R2017a.

The 48 bisyllabic words used for the WRRC test were divided in three lists and were used for each of the three WRRC conditions (i.e., RH, UnSc, and NR conditions). The requirements set for their development was (a) that they represent the Greek language in terms of accentuation, and (b) they do not differ in terms of recognition (for details see Sidiras et al., 2017). Words were ordered within each list according to their inter-Perceptual Center-interval, i.e., from longest to shortest. This ensured that IPI, and therefore tempo of presentation, would not change dramatically across trials, potentially maximizing neural entrainment (see Grube et al., 2010 supplement material, ‘Auditory Testing’).

Two professional musicians (the first author, CS and SE) with 15 years of musical experience were recruited for measuring the words’ P-centers. Musicians are more suitable for this task, as they typically have more finely tuned rhythm perception and temporal processing skills compared to non-musicians (Gaab et al., 2005; Bailey and Penhune, 2010; Bishop-Liebler et al., 2014). WRRC stimuli were comprised of a four-beat sequence (each beat’s duration and frequency being equal to 15 ms and 1.000 Hz respectively) and a following word in noise. While word plus noise characteristics were the same, beat sequence came in three variations corresponding to the three conditions, i.e., Rhythm, Unsynchronized and Non-Rhythm (RH, UnSc, and NR respectively). Note that the length of the stimuli was not affected by the characteristics of the sequence.

The WRRC wordlists consist of 70% of words that are stressed in the first syllable (trochees) versus 30% in the second (iambs) thus the present study findings would be applicable to everyday use of Modern Greek (Iliadou et al., 2006). However, rhythm effects on speech perception should be further investigated to clarify the effects of stress position. Following this rational any potential effect of stress position relates to everyday use of Modern Greek and as any rhythm effect on speech perception should be investigated for both possibilities.

##### Rhythm (RH) condition

The sequence used in RH condition was isochronous and synchronized with the following word (see Figure 1A). That is, all intervals between consecutive beats and P-Centers were the same. Another way to view this is that if the sequence didn’t stop, the word’s 1st and 2nd P-Centers would co-occur with the 5th and 6th beat respectively.

##### Unsynchronized (UnSc) condition

The sequence used in UnSc condition was isochronous and the word was not synchronized to it (see Figure 1B). This was achieved by shortening IBIs by 10%. Hence, intervals between consecutive beats and P-Centers were not the same. Another way to view this is that if the sequence didn’t stop, the word’s 1st and 2nd P-Centers wouldn’t co-occur with the 5th and 6th beat respectively. Instead, 1st and 2nd P-centers would occur between 5th and 6th, and 6th and 7th beats (i.e., at 4.4 and 5.5 IBIs). Shortening, rather than lengthening, was used, as the latter would result in the last beat occurring within the word plus noise, and hence being masked.

##### Non-rhythm (NR) condition

The sequence used in NR condition was not rhythmic (i.e., non-isochronous; see Figure 1C). In order to avoid learning effects which might potentially result in some kind of rhythm perception, several sequences were used in a cyclic order, i.e., first A, then B, then C etc. In all sequences, 30% lengthening and shortening of the sequences’ IBIs were used. This kind of distortion is enough to make a sequence be perceived as non-rhythmic (see Madison and Merker, 2002). Both CS and SE (recruited for the measurement of P-Centers) confirmed that all types of NR beat sequences were ‘heard as being non-rhythmic.’

##### WRRC scoring

Synchronized and unsynchronized rhythm effects are expressed through two derived measures, SREP (Synchronized Rhythm Effect Percentage) and UREP (Un-synchronized Rhythm Effect Percentage) respectively. They are equal to the percentage difference between recognition (i.e., number of correctly identified syllables) in RH vs. NR condition and UnSc vs. NR condition respectively. For example, SREP being equal to 10% means that 10% more syllables are recognized when words are synchronized to rhythm compared to when rhythm is absent. Equally, UREP being equal to 10% means that 10% more syllables are recognized when words are not synchronized to rhythm compared to when rhythm is absent. The benefit of using such derived measures is that they are thought to depend minimally on linguistic ability or cognitive ability (Moore et al., 2010, 2011) and thus reflect a true measure of auditory processing ability.

Positive values in either measure suggest a positive rhythm effect (i.e., better recognition in RH or UnSc condition compared to NR condition), values equal to 0 suggest no effect at all (i.e., same recognition in RH or UnSc conditions compared to NR condition), while negative values suggest a negative rhythm effect (i.e., worse recognition in RH or UnSc condition compared to NR condition). In order to counter-balance for differences in recognition across 1st and 2nd syllable (recognition of the 1st syllable is expected to be significantly better), percentage differences were computed separately, and then the score was calculated as their mean. The formula for calculating SREP was:

$$S\,R\,E\,P=\left[\frac{R\,H\,1_{N}-N\,R\,1_{N}}{N\,R\,1_{N}}\cdot 100\%+\frac{R\,H\,2_{N}-N\,R\,2_{N}}{N\,R\,2_{N}}\cdot 100\%\right]\cdot \frac{1}{2}$$

where RH1_*N*_, RH2_*N*_, NR1_*N*_, and NR2_*N*_ are the number of 1st and 2nd syllables recognized in RH and NR conditions respectively. UREP score was calculated through the same formula, substituting RH1_*N*_, RH2_*N*_ with UnSc1_*N*_ and UnSc2_*N*_ respectively.

### Statistical Analysis

Results followed a normal distribution under the criterion of skewness and kurtosis *z* values ranging between −1.96 and 1.96 (Cramer and Howitt, 2004; Doane and Seward, 2011). Parametric tests were used for statistical analysis, i.e., ANOVA, *t*-Test Pearson correlation, and principal component analysis (PCA). Statistical analysis was executed on SPSS, version 25. Bonferroni corrections where applied whenever needed.

## Results

### WRRC Performance

**FIGURE 2:**
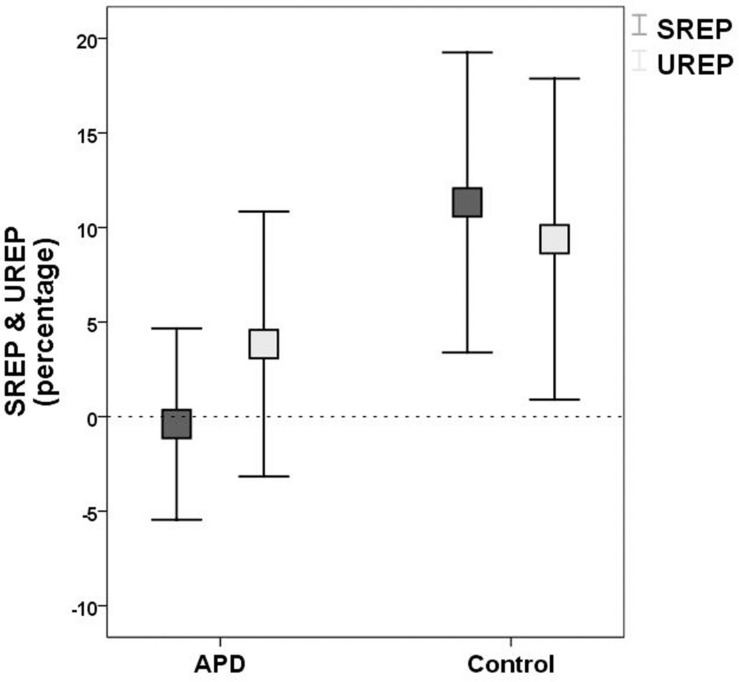
Means and 95% error bars for SREP (dark rectangles) and UREP (bright rectangles) scores for APD and control group respectively. SREP score units are percentages, describing the synchronized rhythm effect on speech in noise recognition. UREP score units are percentages, describing the un-synchronized rhythm effect on speech in noise recognition. Squares represent means. Error bars represent 95% confidence intervals. Dash line (y = 0) represents ‘no effect,’ i.e., recognition not affected by rhythm (see WRRC scoring). SREP, synchronized rhythm effect percentage; UREP, un-synchronized rhythm effect percentage.

Plots of SREP and UREP scores for APD and control children are shown in Figure 2. Descriptive statistics of WRRC raw scores RH1, RH2, UnSc1, UnSc2, NR1, and NR2 (i.e., the number of 1st and 2nd syllables recognized in each condition respectively) for APD and neurotypical children are shown in Table 2. A multivariate 2 by 2 ANOVA was executed (SREP and UREP; APD vs. control group) and revealed significant differences between children with APD and neurotypical children (*F* = 3.392, *p* = 0.03). The two groups differed in SREP scores, APD children scoring significantly lower than neurotypical children (*F* = 6.84, *p* = 0.011,η^2^ = 0.088; mean = −0.4, *SD* = 15.8 vs. mean = 11.3, *SD* = 22.4 respectively). No significant differences were found between groups for UREP scores (*F* = 1.07, *p* = 0.305,η^2^ = 0.015; mean = 3.8, *SD* = 21.9 vs. mean = 9.4, *SD* = 23.9 respectively). A secondary analysis was executed for assessing for differences between condition (i.e., SREP vs. UREP scores) and condition vs. group interaction. A mixed 2 by 2 repeated measures ANOVA in which group was inserted as a between subjects factor revealed no significant differences between condition (*F* =0.285, *p* = 0.545), neither significant interaction (*F* = 2.065, *p* = 0.155).

**TABLE 2:** Means and SDs (in parentheses) of WRRC raw scores RH_*n*_, UnSc_*n*_, and NR_*n*_, i.e., the number of 1st and 2nd syllables recognized in each condition respectively, for APD and neurotypical children.

	**APD group**	**Control group**
**RH**	20.58 (3.58)	21.42 (1.95)
**UnSc**	20.68 (4.04)	20.24 (2.97)
**NR**	20.88 (3.96)	20.88 (2.24)

### Correlations Between Synchronized VS Unsynchronized Rhythm Effects

**FIGURE 3:**
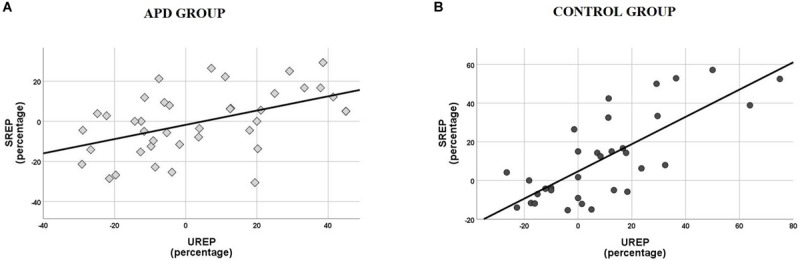
**(A)** Scatterplot of SREP vs. UREP scores for APD group. Correlation is *r* = 0.492, thus the two measures share ∼24% of their total of variance. **(B)** Scatterplot of SREP vs. UREP scores for control group. Correlation is *r* = 0.754, thus the two measures share ∼57% of their total of variance. SREP, synchronized rhythm effect percentage; UREP, un-synchronized rhythm effect percentage.

Scatterplots of SREP vs. UREP scores for APD and control children shown in Figure 3, suggest a correlation between synchronized vs. unsynchronized rhythm effects on speech recognition. That is, positive and large effects of synchronized rhythm tend to coincide with positive and large effects of un-synchronized rhythm, and vice versa. Pearson test revealed a significant correlation between SREP vs. UREP scores within both the APD (*r* = 0.492, *p* = 0.001) and the control group (*r* = 0.754, *p* < 0.001). The two effects share a total of ∼24% variance within the APD group, while the respective shared variance is ∼57% within the control group. A single sided test (Eid et al., 2011) revealed that the difference of shared variance between groups was statistically significant (*z* = 1.805, *p* = 0.036).

### WRRC VS Auditory Processing and Cognition

Correlation between SREP and UREP scores vs. performance in the test battery delivered in our clinic (SinB, GIN, RGDT, DD, PPS, DPS, SAA, Digit Span) was assessed through Pearson test. For the control group the analysis was executed for the speech in noise recognition test (SinB) and the two cognitive tests (sustained auditory attention, SAA; working memory, Digit Span), as the rest of the tests were not delivered (see Table 1). Regression analysis would be more appropriate for assessing correlation between WRRC measures vs. multiple tests outcomes. However, as APD children were randomly selected from a clinic, about half of them completed the SAA, GIN, DD, PPS, and DPS tests in a non-overlapping manner (i.e., a child might complete SAA but not DD). Hence, the number of children that complete all tests are very low, rendering the multiple regression non-applicable. This is matter of clinical evaluation directed toward medical history taken. All correlation analyses’ results are shown in detail in Table 3.

**TABLE 3:** Correlation analysis results between SREP and UREP scores vs. SinB, Digit Span, SAA, GIN, RGDT, DD, PPS, and DPS performance for APD and control group.

	**APD group**		**Control group**	
	**SREP**	**UREP**	**SREP**	**UREP**
SinB	−0.196(0.239)	0.052 (0.757)	−0.017(0.924)	−0.090(0.620)
Digit Span	0.341 (0.095)	0.152 (0.469)	−0.033(0.877)	−0.122(0.569)
SAA	0.464* (0.026)	0.219 (0.315)	−0.081(0.669)	−0.025(0.896)
GIN	−0.154(0.483)	−0.324(0.131)	−	−
RGDT	−0.417* (0.020)	−0.559** (0.001)	−	−
DD	0.364 (0.088)	0.427* (0.042)	−	−
PPS	0.810** (< 0.001)	0.362 (0.168)	−	−
DPS	0.591* (0.026)	0.886** (<0.000)	−	−

*Significant at the 0.05 *p*-value, **Significant at the 0.01 *p*-value.

**FIGURE 4:**
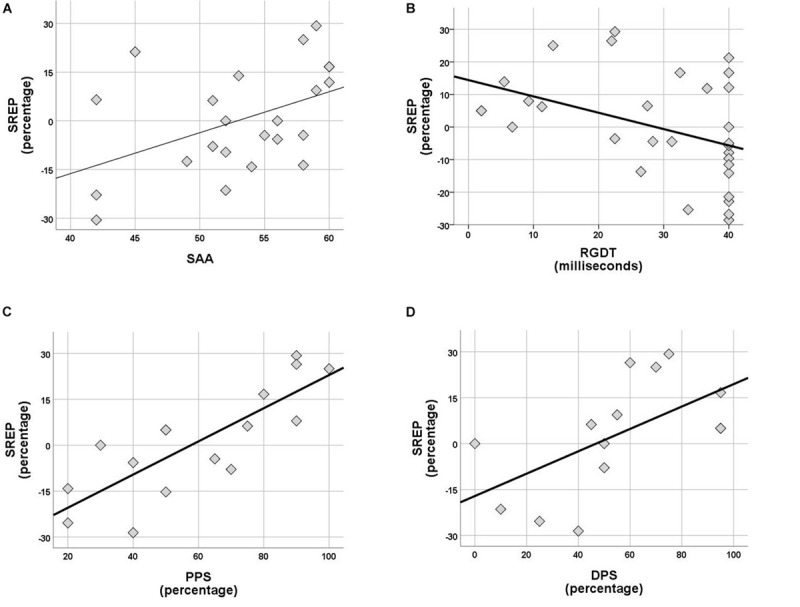
**(A–D)** Scatterplots of SREP scores vs. SAA, RGDT, PPS, and DPS respectively, for the APD group. Correlations are *r* = 0.464, *p* = 0.026, *r* = –0.417, *p* = 0.020, *r* = 0.810, *p* < 0.001 and *r* = 0.591, *p* = 0.026. SREP, synchronized rhythm effect percentage; SAA, sustained auditory attention; PPS, pitch pattern sequence; DPS, duration pattern sequence.

**FIGURE 5:**
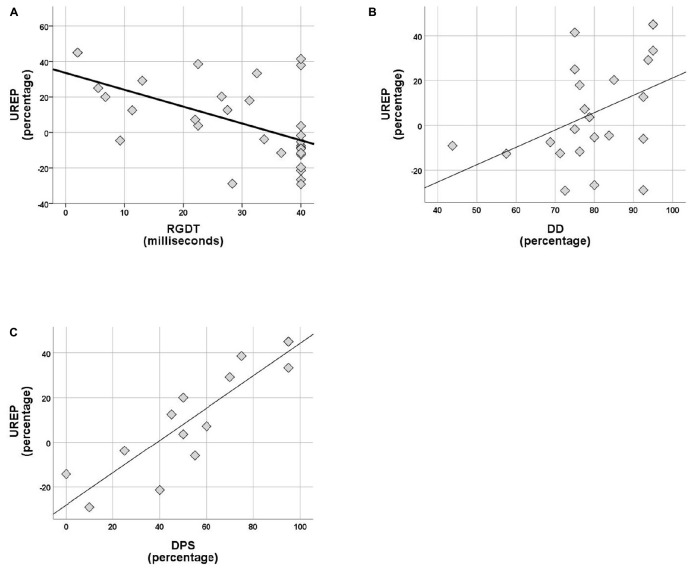
**(A–C)** Scatterplots of UREP scores vs. RGDT, DD, and DPS respectively, for APD group. Correlations are *r* = –0.559, *p* = 0.001, *r* = 0.427, and *r* = 0.886 respectively. UREP, un-synchronized rhythm effect percentage; DD, dichotic digits; DPS, duration pattern sequence.

Within the control group no correlations were observed between rhythm effect and the rest of the tests delivered (SinB, SAA, and Digit Span). Within the APD group, SREP correlated with sustained auditory attention (SAA, *r* = 0.464, *p* = 0.026), one temporal resolution test (RGDT, *r* = −0.417, *p* = 0.020), and both pattern sequencing tests (PPS, *r* = 0.810, *p* < 0.001; DPS, *r* = 0.591, *p* = 0.026; see Figure 4). UREP correlated with one temporal resolution test (RGDT, *r* = −0.559, *p* = 0.001), dichotic listening (DD; *r* = 0.427, *p* = 0.42) and one pattern sequencing test (DPS; *r* = 0.886, *p* < 0.001; see Figure 5). As multiple correlation tests were executed, Bonferroni correction was applied, yielding the significance level of 0.00625. Correlations that remained significant were SREP vs. PPS, UREP vs. RGDT and UREP vs. DPS. However, note that in this case Bonferroni correction may easily result in type II errors as *r* values must be higher than 0.48, 0.55, and 0.7 for RGDT, SAA, and DPS respectively for the correlation to remain significant given the sample size available for each comparison.

To further assess the correlations between WRRC test and the rest of the auditory tests, a PCA analysis was executed. SREP and UREP were fed into the model which yielded one factor with eigenvalue greater than 1 (equal to 1.492). The extracted WRRC factor was then fed into Pearson analysis with the rest of the test, yielding significant correlations with RGDT (*r* = −0.545, *p* = 0.002), DD (*r* =0.457, *p* =0.023), PPS (*r* = 0.639, *p* = 0.008), and DPS (*r* = 0.807, *p* < 0.001). As multiple correlation tests were executed, Bonferroni corrections were applied, yielding the significance level of 0.0083. Correlations which remained significant were the WRRC factor vs. RGDT, PPS, and DPS.

### SINB, Sustained Auditory Attention, and Working Memory Performance

Auditory processing disorder children scored significantly worse in SinB and working memory (Digit Span) compared to neurotypical children (SinB: mean = 1.1, *SD* = 1.4, vs. mean = 0.57, *SD* = 0.6, *F* = 4.1, *p* = 0.46; Digit Span: mean = 8.9, *SD* = 2.37 vs. mean = 11.4, *SD* = 2.52, *F* = 12.37, *p* =0.001 respectively). There were no significant differences between groups for Sustained Auditory Attention (mean = 53.2, *SD* = 5.9 vs. 54.7, *SD* = 5.1, for APD and neurotypical children respectively, *F* = 0.987, *p* = 0.325).

## Discussion

In the present study, the effect of auditory rhythm on synchronized and unsynchronized word in noise recognition (referred to as ‘synchronized rhythm effect’ and ‘unsynchronized rhythm effect’ respectively) was assessed for APD and typically developing primary school children via the WRRC test (see also Sidiras et al., 2017). These effects were then compared to other aspects of auditory perception as examined in everyday clinical practice, and cognition (sustained auditory attention and working memory).

The primary hypothesis of the study was partially confirmed as our results supported the absence of synchronized effects in children with APD but were unclear for unsynchronized effects. The two secondary ones were confirmed as (i) for APD children rhythm effects were correlated to performance in half of auditory processing clinical tests used in our clinic, and (ii) cognition was found to be a factor of rather small importance, as sustained auditory attention accounted for ∼20% of the variance for the synchronized (but not unsynchronized) rhythm effect for the APD group (but not for the typically developing group). Working memory, as measured by Digit Span test, did not correlate with rhythm effects in both groups.

### Rhythm Effect in APD Children

For children with APD no synchronized rhythm effects were observed, though results were unclear for unsynchronized rhythm effects as revealed by *post hoc* analysis (in which effects were assessed separately for SREP and UREP). The absence of EEG data doesn’t permit us to draw direct conclusions on a neurobiological level. However, given the amount of evidence supporting the link between Dynamic Attending and neural entrainment (see reviews Obleser and Kayser, 2019; Malaia and Wilbur, 2020), we argue that our results may indicate deficits in neural entrainment. Drawing conclusions for neurobiology from psychometric results is not uncommon (see Bedoin et al., 2016, 2018). Moreover, this interpretation is in line with evidence from EEG studies for deficits in neural entrainment linked to deficits in sensory processing in other neurodevelopmental disorders, i.e., dyslexia (Colling et al., 2017; Di Liberto et al., 2018), and ADHD (Calderone et al., 2014; Puyjarinet et al., 2017; also see Lakatos et al., 2019).

We interpret the absence of rhythm effects as an indication of a deficit in the neural entrainment mechanism. Note that this result does not suggest necessarily the absence of neural oscillations in APD children. According to DAT, the phase of the oscillation when the stimulus arrives plus the value around which processing power oscillates are the determining factors of the processing power. DAT assumes that entrainment is always in phase with the stimuli’s rhythm. This assumption seems to work for a neurotypical population, but it may not be true for APD children. Small fluctuations in phase across trials are indeed observed (see phase coherence; Busch et al., 2009; Breska and Deouell, 2017), and one could expect these fluctuations to be larger in the APD population. This is further supported by the fact that large phase fluctuations across trials are correlated to poor performance (Busch et al., 2009; Breska and Deouell, 2017), which is one of the characteristics of APD children for auditory tests. Thus, the absence of rhythm effects in APD children observed here is consistent with both neural entrainment being absent and/or low phase coherence across trials in neural entrainment.

### Rhythm Effect vs. Auditory Processing and Cognition

For the APD group, both synchronized and unsynchronized rhythm effects correlated with half of the auditory processing tests, all of them being non-verbal, with the exception of Dichotic Digits test (stimuli are digits). This result seems counterintuitive given that rhythm effects were absent in the APD children group. However, given the variability of their scores (*SD* = 15.8 and *SD* = 21.9 for SREP and UREP respectively), this doesn’t rule out effects at the individual level. Whether priming effects are of relevance in individual cases with APD is a debatable issue that we cannot adequately address in this study.

The correlations observed suggest that deficits in other facets of non-verbal auditory processing (as examined in clinic) tend to co-exist with deficits in rhythm effects. This finding also strongly suggests that even though the stimuli to be recognized in WRRC are verbal (i.e., words), the rhythm effect reflects mostly a non-verbal auditory processing ability, which is expected, since the effect is a derived measure (Moore et al., 2010, 2011).

Rhythm effects correlated strongly with performance in the two pattern sequencing tests (Pitch Pattern Sequence and Duration Pattern Sequence; Musiek, 1994), sharing large portions of variance (namely 65.1, 34.9, and 78.5%). Pitch and duration processing are arguably not the best candidates for the common underlying factors. Instead, a single factor present in both tests, namely auditory temporal ordering processing, offers a simpler explanation for correlations of rhythm effects with both of them. This view in line and furtherly supports Roux and Uhlhaas (2014), according to whom theta-band (∼4 Hz) neural oscillations are involved in the temporal organization of working memory items. Theta-band neural oscillations are arguably implicated in both the WRRC test (as a result of the isochronous beat sequence) and PPS and DPS tests (as a result of the temporal organization required for the task, i.e., labeling a sequence of three presented tones in the right order, in terms of pitch and duration respectively).

As regards cognition, sustained auditory attention predicted only a part of rhythm effects, i.e., ∼22% of the synchronized condition for the APD group, while no correlation was observed for the neurotypical group. The WRRC does not require attentional resources, suggesting that the rhythm effect relies mostly on a lower level mechanism. Working memory tested by Digit Span did not correlate at all with rhythm effects for either group. This is not necessarily in conflict with the interpretation concerning working memory given above for the correlation with the pattern sequencing tests. In both PPS and DPS, working memory is loaded with sensory information, i.e., pitch and duration respectively, rather than verbal as in the case of Digit Span, and this kind of information is not processed in the same way in working memory (Wilsch and Obleser, 2016). Furtherly, sensory, rather than verbal processing correlating with impairments in APD children (here deficit in rhythm effects), fits better with the view that APD concerns mainly impairments in lower levels of auditory perception and less with cognition (American Speech-Language-Hearing Association [ASHA], 2005; American Academy of Audiology [AAA], 2010; Iliadou et al., 2017; Stavrinos et al., 2018; Sidiras et al., 2019).

### Synchronized vs. Unsynchronized Rhythm Effect

Correlation between synchronized vs. unsynchronized rhythm effect was observed in both groups, though it was significantly larger for the neurotypical group. The shared variance was ∼24 and ∼57% for the APD and the neurotypical group respectively. This suggests that there is a large portion of unsynchronized rhythm effect variance that cannot be explained in terms of synchronized rhythm effect. Furtherly, the factors that determine the unsynchronized rhythm effect (which differ from the factors determining the synchronized effect) have a larger influence on APD children compared to neurotypical ones (∼76 vs. ∼43% respectively). This larger variability in APD is in line with the complexity and the heterogeneity that characterizes the disorder (American Speech-Language-Hearing Association [ASHA], 2005; American Academy of Audiology [AAA], 2010; British Society of Audiology, 2018).

### Rhythm Effects in Typically Developing Children

Our results suggest that rhythm enhances processing power of the recognition of words in noise for children, compared to when rhythm is absent. This may be explained by neural entrainment. That is, the preceding rhythmic beat sequence in the WRRC stimuli resulted in neural entrainment (Overath et al., 2015; Zoefel et al., 2018), and neural entrainment resulted in enhancement in processing power, i.e., word recognition. The effect was found for both synchronized and unsynchronized words, though it was a little smaller for the latter (∼11 vs. ∼9% better recognition respectively). This difference, though too small to be statistically significant, is in line with previous results for WRRC in Sidiras et al. (2017).

Though synchronized rhythm effect is predicted by DAT, unsynchronized rhythm effect is neither a prediction, nor disproves DAT, but rather expands previous findings on the enhancement of syntax processing when preceded/primed by auditory rhythm (Przybylski et al., 2013; Kotz and Gunter, 2015). For unsynchronized rhythm effect to be present, the oscillations in processing power must take place above the processing power for resting state, i.e., when neural entrainment is absent. Otherwise, unsynchronized syllables would co-occur with processing power equal or less than the one in resting state, resulting in no or negative unsynchronized rhythm effect. DAT posits periodic fluctuations in processing power, but does not predict its relation to resting state. In any case, our results suggest that for the case of the WRRC test, the mean value of these fluctuations must be close to SREP and UREP scores, i.e., ∼10% higher than the resting state in terms of word recognition for typically developing children.

### Implications for Future Research

This is the first study assessing for effects of rhythm on speech in noise recognition for APD children. APD assessment typically includes speech in noise recognition, temporal sequencing, temporal resolution and dichotic listening tests, which arguably do not capture all manifestations of APD. Further, the present study confirms and furtherly expands on previous research on rhythm perception of this population (Olakunbi et al., 2010; Scheffner et al., 2017; Sidiras et al., 2019). The WRRC wordlists consist of 70% of words that are stressed in the first syllable (trochees) versus 30% in the second (iambs) thus the present study findings would be applicable to everyday use of Modern Greek (Iliadou et al., 2006). However, rhythm effects on speech perception should be further investigated to further clarify the effects of stress position. Here, we propose a set of open questions related to auditory rhythm for future research.

– What is the nature of the rhythm effect deficit? We offered above two alternative (but not mutually exclusive) proposals, i.e., neural entrainment being absent and low phase coherence.
– How is the deficit in rhythm effect manifested in everyday listening situations for APD children?
– Do APD children present with the typical 4 Hz peak sensitivity?
– Is syllable parsing (see Giraud and Poeppel, 2012) correlated to rhythm effect?
– Can music/rhythm training restore deficits in rhythm effects?

## Data Availability Statement

The datasets generated for this study are available on request to the corresponding author.

## Ethics Statement

The studies involving human participants were reviewed and approved by Ethics and Bioethics Committee of the Aristotle University of Thessaloniki. Written informed consent to participate in this study was provided by the participants’ legal guardian/next of kin.

## Author Contributions

CS, VI, D-EB, and IN conceived and designed the study, made substantial contribution to the data interpretation, critically revising the drafted manuscript, final approval of the version to be published, and agreement to be accountable for all aspects of the work in ensuring that questions related to the accuracy or integrity of any part of the work are appropriately investigated and resolved. CS performed the experiment and statistical analysis, and wrote the first draft of the manuscript. VI and D-EB critically revised the drafted manuscript.

## Conflict of Interest

The authors declare that the research was conducted in the absence of any commercial or financial relationships that could be construed as a potential conflict of interest.
